# Government digital policy breaks the mystery of “limited participation” in China's home finance market

**DOI:** 10.1038/s41598-023-47372-6

**Published:** 2023-11-19

**Authors:** Lu Xing, DongHao Han, Hui Xie

**Affiliations:** 1 School of Business, Yunnan University of Finance and Economics, 237 Longquan Road, Wuhua District, Kunming, 650021 Yunnan China; 2 School of Mathematical Sciences, Fudan University, Shanghai, 200433 China; 3 School of Business, Kunming University of Science and Technology, Kunming, 650500 Yunnan China

**Keywords:** Socioeconomic scenarios, Psychology and behaviour

## Abstract

This study uses a two-step approach to construct a multi-period double-difference model and introduces a quasi-natural experiment of the Broadband China pilot policy to investigate whether household financial market participation at the urban level is affected by the digital economy, which is significant for promoting Chinese households' shift from savings to investment and alleviating the long-standing problem of insufficient household financial market participation in China. In terms of direct impact, the digital economy increases the household financial market participation rate of urban residents by 3.26%, and increases the financial market participation rate of highly financially literate households by 2.14%; in terms of indirect impact, the development of the digital economy increases the total number of household smart Internet devices by 8.27%, and similarly increases the attention to household financial information by a significant 4.22%, which further positively influences the household financial market participation rate. This paper also evaluates the individual and regional differences of the digital economy on household financial market participation, and the estimated causal effect of the digital economy on household financial market participation is purer, which expands the scope of research on the digital economy and household financial market participation, and provides a certain reference basis and policy inspiration for the government to promote the construction of the digital economy.

## Introduction

The macroeconomic recovery in the post-epidemic era has caused a gradual shift in Chinese residents' asset allocation from risk-free broad asset classes to risky financial assets. Financial asset allocation is now entering an accelerated period and residents' effective participation in financial markets is crucial to the long-term sound appreciation of the family wealth^1–4^. The advancement of the digital economy provides effective support for household participation in financial markets by broadening access to financial literacy and enhancing the convenience of financial investment for residents^5,6^. Aiming to analyse the implications of the digital economy for household participation in financial markets, scholars have quantified the digital economy in terms of both the level of Internet development and the level of digital financial development^7^. However, no uniform conclusions have been reached. Even though several scholars have discussed the effect of digital economy policies^8–10^, existing studies have ignored how these policies affect household financial market participation rates at the city level. As a result, the mechanism and heterogeneity analysis of the impact of the digital economy on household financial market participation rates needs to be further investigated. At the micro level, this paper is important for reducing household financial vulnerability, increasing household income diversity, and thus improving quality of life and welfare. At the macro level, guiding the transformation of residents' savings to the investment side, helping in expanding domestic demand, promoting the sound development of China's financial market, and achieving the strategic goal of a commonwealth is important for the government.

The latest edition of the China Household Finance Survey (CHFS) published in 2019 shows that Chinese households are not active in the financial market, with real estate assets accounting for 66.3% and financial assets (including stocks, bonds, funds and wealth management products) accounting for only 12.8%. In terms of broad asset allocation, real estate accounts for only 15% of U.S. households and 46% in Europe, compared to Chinese households' over-investment in real estate. In terms of financial asset allocation, the proportion of financial assets of American households is as high as 51%, and that of European households is 28%, while the proportion of financial asset allocation of Chinese households is at a relatively low level in the international arena. It is worth noting that in recent years, China's booming digital economy has become a possible means to address the mystery of "limited participation" in China's household financial market. Given that digital infrastructure has a positive impact on enhancing the financial literacy of the population^11^, and that broadband infrastructure is one of the key components of the digital economy^12^, and that broadband service policies can effectively penetrate in free economies throughout the world^13^, the aforementioned literature serves as the basis for the investigation in this paper.

On the one hand, the "Broadband China" policy aims to optimize and expand the construction of Internet infrastructure for broadband applications, and on the other hand, the "Broadband China" policy also includes the enhancement of residents' ability to use the Internet, the cultivation of digital consumption habits, and the promotion of the digital transformation of industries, therefore, the "Broadband China" policy is a good proxy for the development of the digital economy, and is mostly used in the literature to assess the impact of the digital economy^14^. This study explores the impact of the digital economy on the financial market participation rate of Chinese households by using the "Broadband China" pilot policy as a quasi-natural experiment as well as a two-step method to construct a multi-period double difference model based on Chinese household financial survey data. The following are the possible marginal contributions of our study: first, this paper introduces a quasi-natural experiment on the basis of the perspective of digital economy development to determine the causal effect of the digital economy on the household financial market participation rate. Second, this paper uses a two-step approach to construct a multi-period double-difference model that controls for individual-level characteristics without losing individual-level information and integrates information on household financial market participation at the prefecture level. The estimated household financial market participation rate at the city level is more statistically representative. Finally, this paper likewise assesses the regional differences and individual differences in household financial market participation rates that are affected by the digital economy while verifying the influence mechanism from two paths: information transmission and financial information concern, which broadens the scope of the study and deepens the paper’s policy connotation.

The following parts of this paper are organised as follows: the second part consists of the policy background; the introduction of data, variables, and models is covered in the third part; the fourth part discusses the main empirical results, robustness test, heterogeneity test, and mechanism discussion; the fifth part involves the research conclusion.

## Background

The strategy implementation plan of "Broadband China" was released by the State Council in August 2013, and the Ministry of Industry and Information Technology and the National Development and Reform Commission released the list of 117 "Broadband China" demonstration cities (clusters) in three batches from 2014 to 2016. The objective is to optimize and upgrade broadband networks and reinforce the application of broadband networks.Since the implementation of the "Broadband China" strategy, China has made significant progress in digital infrastructure construction. According to the Ministry of Industry and Information Technology's 2021 Communications Industry Statistical Bulletin, by the end of 2021, the number of China's Internet broadband access ports reached 1.018 billion, up from fewer than 15 million at the start of the twenty-first century.

Several scholars have investigated various dimensions based on the quasi-natural experiment of the implementation of the Broadband China policy. At the consumption level, the implementation of the "broadband China" policy has a considerable positive impact on rural household consumption and electricity consumption^15,16^; at the firm level, the implementation of the "broadband China" policy aids in improving firm productivity and concentration^17,18^ and increasing the number of mergers and acquisitions^19^; at the level of green sustainable development, the implementation of "broadband China" policy further improves the effectiveness of green innovation in China through its impact on green technology innovation^20,21^; at the financial market level, the implementation of the "broadband China" policy has successfully mitigated the risk of stock price collapse^22^ and played a positive role in the spread of digital inclusive finance^23^. However, no scholars have evaluated the impact of the implementation of the "broadband China" policy at the level of household financial market participation.

Under the assumption of information asymmetry, investors lacking information have negative returns relative to the total market return, whereas investors with information can buy low and sell high to gain additional returns, which comes from information profit. An information-asymmetric market can make it difficult for households to engage in financial markets, which might cause some households to avoid using financial services. Through the development of network infrastructure and the optimisation of digital infrastructure, the "broadband China" policy's implementation reduces the obstacles to financial market participation caused by information asymmetries, giving the implementation of the "Broadband China" policy in the pilot cities an opportunity to explore the impact of the digital economy on household financial market participation.

## Methodology

### Data

This paper examines the implementation of the Broadband China policy as a quasi-natural experiment by using data from the China Household Finance Survey (CHFS) for 2013, 2015, 2017, and 2019 to ascertain whether the digital economy can enhance the likelihood of household financial market participation in China. The "Broadband China" policy data were compiled from the list of selected "Broadband China" demonstration cities from 2014 to 2016 published by the Ministry of Industry and Information Technology of the People's Republic of China. The China Household Finance Survey data use modern sampling methods, a computer-assisted survey system (CAPI), along with other survey techniques and management tools to control sampling and non-sampling errors, and the data are representative and of high quality, which is the most extensively employed micro household finance data. The data of other prefecture-level cities used in this paper primarily comes from the China City Statistical Yearbook.

### Empirical model

In this paper, the multi-period double difference method is used for estimation. If respondents' participation in financial markets is the explained variable, direct individual-level estimation encounters two major issues: first, Logit and Probit models, which are typically utilised for regressions on dichotomous variables, do not apply in double difference estimation, and marginal effects calculated based on regression coefficients are even less meaningful; Second, even switching to a linear probability model for estimation does not yield an intuitive explanation of the influence of a digital economy on household financial market participation^24^. In light of this, a two-step approach is used for the estimation in this paper^25^. In particular, this study first integrates data on household financial market participation to the prefecture-level controlling for individual characteristics and then performs double-difference estimation using prefecture-level data, in contrast to direct estimation with individual-level data, estimation with prefecture-level data allowing for intuitive interpretation of the estimated coefficients without losing individual-level information. Moreover, due to the fact that individual-level characteristics are controlled for in the first estimation step, the city-level household financial market participation estimated from this is more statistically representative. The model is estimated in the first step as:

1 $$Fin\_Par_{it} = \beta_{1} gender_{it} + \beta_{2} age_{it} + \beta_{3} edu_{it} + \beta_{4} marriage_{it} + \; \beta_{5} health_{it} + \beta_{6} Finit_{it} + \beta_{7} house_{it} + \beta_{8} RA_{it} + \;\beta_{9} Ln\_assets_{it} + \beta_{10} Ln\_income_{it} \mathop \sum \limits_{jt} FPR_{jt} city_{ijt} + \mu_{it}$$

In the above equation, subscript $i$ represents the individual, subscript $j$ denotes the city, subscript $t$ indicates the year, ${Fin\_Par}_{it}$ indicates whether individual $\mathrm{i}$ took part in the financial market in year $\mathrm{t}$. If individual $\mathrm{i}$ participated in the financial market in year $t$, the variable is assigned a value of 1. If individual $i$ did not participate in the financial market in year $t$, the variable is assigned a value of 0. $gender_{it}$, $age_{it}$, $edu_{it}$, $marriage_{it}$, $health_{it}$, $Finit_{it}$, $house_{it}$, $RA_{it}$, ${\text{Ln}}\_assets_{it}$, ${\text{Ln}}\_income_{it}$ represents the individual-level control variables, $city_{ijt}$ is the city dummy variable, and the dummy variable coefficient $FPR_{jt}$ for each city is obtained after performing a no-intercept regression, which is the city-level household financial market participation rate after excluding individual differences. The individual level variables are interpreted as shown in Table 1:

**Table 1:** Definition of individual-level variables.

Variable definition	
$Fin\_Par_{it}$	Whether the household is involved in the financial market, involved = 1, not involved = 0
$gender_{it}$	Female = 0; Male = 1
$age_{it}$	Age is rounded to the nearest whole number
$edu_{it}$	Resident's education level, no schooling = 1; elementary and junior high school = 2; high school and junior college = 3; college = 4; undergraduate and graduate (including master's and doctoral) = 5
$marriage_{it}$	Married = 1; Unmarried = 0
$health_{it}$	Self-evaluation of health, healthy = 1; unhealthy = 0
$Finit_{it}$	Financial literacy, financial literacy indicators are constructed to measure financial literacy from three aspects: financial information, financial knowledge, and financial ability, and are analyzed using the iterative principal factor method. The construction indicators are detailed in the Appendix 1
$house_{it}$	Whether there is a house,Yes = 1;No = 0
$RA_{it}$	Risk attitude, Five bands of high risk and high return, slightly high risk and slightly high return, average risk and average return, slightly low risk and slightly low return, and unwillingness to take risk, with values ranging from 5 to 1
${\text{Ln}}\_assets_{it}$	Total Assets, the logarithm of the household's total assets for the year
${\text{Ln}}\_income_{it}$	Total income, the logarithm of the household's total income for the year

The city-level household financial market participation calculated in the first step is used in the second estimation step together with other data to create an unbalanced panel data and is regressed using a multi-period double difference model with the following estimated model:

2 $${\text{FPR}}_{jt} = \gamma_{0} + \gamma_{1} Policy_{jt} + \gamma_{2} GDP_{jt} + \gamma_{3} FD_{jt} + \gamma_{4} Salary_{jt} + \gamma_{5} CONSP_{jt} + \gamma_{6} UR_{jt} + \gamma_{7} FI_{jt} + \gamma_{8} TIE_{jt} + \gamma_{9} FE_{jt} + \gamma_{10} SEC_{jt} + \gamma_{11} TER_{jt} + \lambda_{j} + \eta_{t} + \varepsilon_{jt}$$

$j$ subscript denotes the city, $t$ subscript represents the year,${\text{FPR}}_{{{\text{jt}}}}$ reflects the city-level household finance market participation estimated in the first step, $Policy_{jt} = treat_{j} \times after_{t}$. Where $treat_{j}$ is a group dummy variable, $treat_{j} = 1$ if city ${\text{j}}$ is selected as a "Broadband China" demonstration city, otherwise $treat_{j} = 0$; $after_{t}$ is a time dummy variable, $after_{t} = 0$ before city ${\text{j}}$ is selected as a "Broadband China" demonstration city, and $after_{t} =$ 1 in the year and after.$FD_{jt}$, $GDP_{jt}$, $Salary_{jt}$, $CONSP_{jt}$, $UR_{jt}$, $FI_{jt}$, $TIE_{jt}$, $FE_{jt}$, $SEC_{ij}$, $TER_{it}$ are the control variables, $\lambda_{j}$ is the city fixed effect, and $\eta_{t}$ is the year fixed effect. In order to address possible heteroskedasticity in the model, robust standard errors are applied in the regressions. $\gamma_{1}$ denotes the effect of the digital economy on household financial market participation rates, which is the focus of this paper's research. The city level variables are interpreted as shown in Table 2:

**Table 2:** Definition of city-level variables.

Variable definition	
${\text{FPR}}_{{{\text{jt}}}}$	Household financial market participation rates, excluding individual differences at the city level
${\text{Policy}}_{{{\text{jt}}}}$	Pilot policy interaction term, cross product of group dummy variable and time dummy variable
${\text{FD}}_{{{\text{jt}}}}$	Financial development, Commercial bank loan-to-deposit ratio = financial loan balance/financial deposit balance
${\text{GDP}}_{{{\text{jt}}}}$	Total output—intermediate inputs
${\text{Salary}}_{{{\text{jt}}}}$	Income per unit, total employee wages/average number of employees
${\text{CONSP}}_{{{\text{jt}}}}$	Consumption level, total social retail consumption/GDP
${\text{UR}}_{{{\text{jt}}}}$	Urbanization rate, urban population/total population
${\text{FI}}_{{{\text{jt}}}}$	Scale of investment, fixed asset investment/GDP
${\text{TIE}}_{{{\text{jt}}}}$	Foreign trade dependence, total exports and imports/GDP
${\text{FE}}_{{{\text{jt}}}}$	Fiscal concentration, fiscal expenditure/GDP
${\text{SEC}}_{{{\text{jt}}}}$	Percentage of secondary industry, value added of secondary industry/GDP
${\text{TER}}_{{{\text{jt}}}}$	Percentage of tertiary sector, value added of tertiary industry/GDP

## Empirical results

### Descriptive statistics

The findings of descriptive statistics are depicted in Table 3. In this paper, the samples with the interaction item of the "Broadband China" pilot policy assigned to 1 are classified as the treatment group, while the other samples are categorised as the control group. The original judgment that the digital economy is conducive to increasing the probability of household participation in financial markets is supported given that households in the intervention group are more likely to participate in financial markets than those in the control group. In addition, the differences in the values of most control variables between the treatment and control groups during the sample period are small, which to a certain extent explains the exogeneity of the "broadband China" policy.

**Table 3:** Descriptive statistical analysis.

	Control	Treat	Total
FPR	0.151	0.193	0.167
	(0.0472)	(0.0694)	(0.0605)
Policy	0	0.663	0.261
	(0)	(0.474)	(0.439)
FD	0.0898	0.0759	0.0844
	(0.0612)	(0.0230)	(0.0502)
GDP	0.0845	0.155	0.112
	(0.0527)	(0.101)	(0.0829)
Salary	0.329	0.372	0.346
	(0.0936)	(0.114)	(0.104)
CONSP	0.403	0.400	0.401
	(0.123)	(0.110)	(0.118)
UR	0.529	0.656	0.580
	(0.119)	(0.142)	(0.143)
FI	0.840	0.798	0.823
	(0.346)	(0.348)	(0.347)
TIE	0.124	0.289	0.190
	(0.252)	(0.414)	(0.336)
FE	0.216	0.165	0.196
	(0.0895)	(0.0589)	(0.0826)
SEC	43.21	44.69	43.80
	(10.09)	(9.338)	(9.814)
TER	42.84	47.89	44.85
	(8.381)	(10.61)	(9.650)
N	416	270	686

### Baseline estimate

Table 4 presents the findings of the benchmark regressions on the effect of the digital economy on the probability of household financial market participation; column (1) is univariate, column (2) accounts for city and year effects, column (3) further incorporates other control variables at the city level. According to the results, the digital economy raises the likelihood of household financial market participation in China by 2.64%, and its development stimulates residents to participate in financial markets and make household risky financial asset allocations.

**Table 4:** Baseline estimate.

	FPR		
	(1)	(2)	(3)
Policy	0.0415***	0.0241***	0.0264***
	(0.0039)	(0.0048)	(0.0051)
GDP			0.0576
			(0.0429)
FD			0.0186
			(0.0303)
Salary			0.1327**
			(0.0532)
CONSP			0.0527**
			(0.0253)
UR			0.0332
			(0.0451)
FI			0.0001
			(0.0058)
TIE			-0.0067
			(0.0077)
FE			0.0982
			(0.0597)
SEC			0.0006
			(0.0011)
TER			0.0004
CityFE	No	Yes	Yes
YearFE	No	Yes	Yes
N	686	686	686
r2_a	0.6032	0.6292	0.6310

Standard errors in parentheses.

*p < .10, **p < .05, ***p < .01.

### Robustness tests

#### Parallel trend test

**Figure 1:**
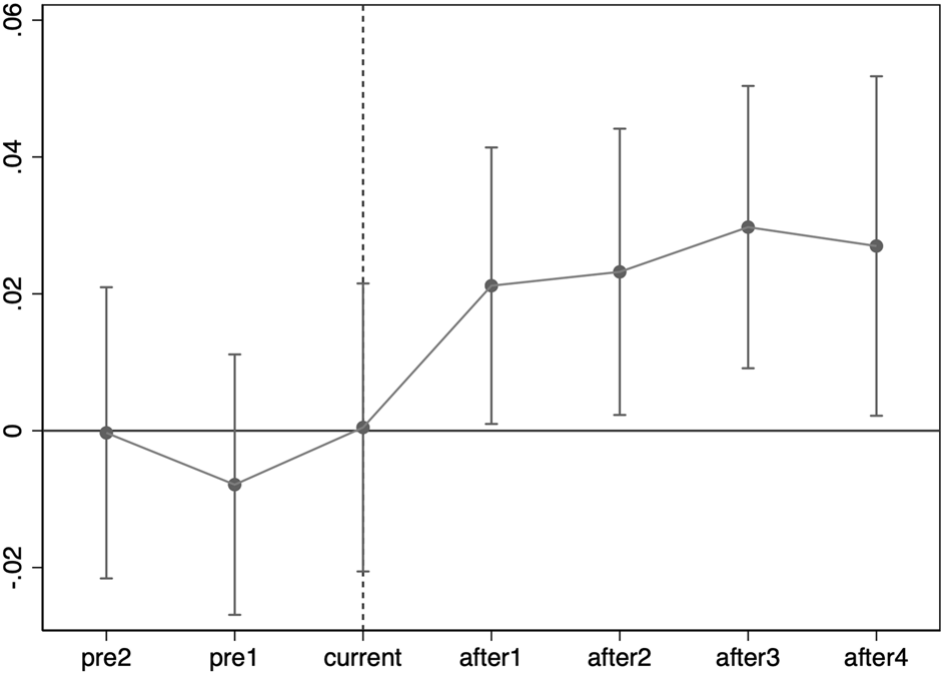
Parallel trend test.

As shown in Fig. 1, the coefficients of pre2 and pre1 are not significant prior to implement the "broadband China" policy, which points out that the outcome variables have the same trend in the control and treatment groups, and the control and treatment groups satisfy the hypothesis of parallel trends. Meanwhile, the probability of financial market participation of Chinese households in the year of policy implementation is not affected owing to the fact that the policy implementation effect has a certain lag and the "broadband China" policy is implemented in September or October of the year. The coefficients of after1, after2, after3, and after4 are all significant after the implementation of the policy, demonstrating that the growth of the digital economy has greatly boosted the probability of household financial market participation.

#### Placebo test

**Figure 2:**
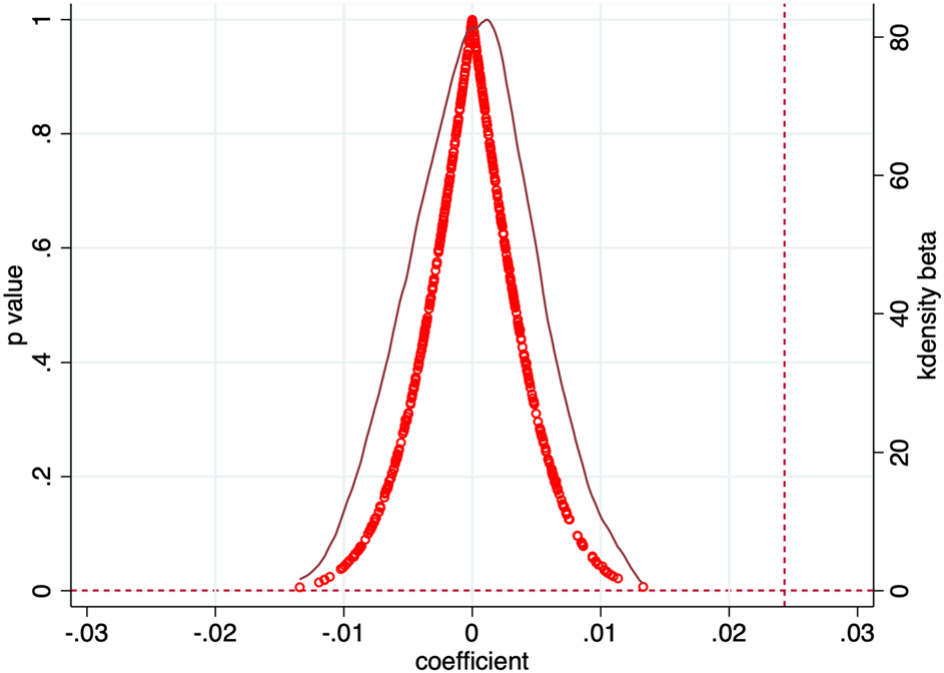
Placebo test. The blue horizontal dotted line above indicates areas with *p*-values greater than 0.1 and other areas with p-values less than 0.1; the red vertical dotted line indicates where the true coefficient of 0.243 is located.

This paper constructs a randomised experiment at both the pilot city and implementation time levels by randomly selecting the pilot cities of Broadband China and generating the implementation time of the policy at random. The randomisation process is repeated 500 times and the regression is performed 500 times. Figure 2 shows the distribution of regression coefficients using 500 spurious experiments. These coefficients are concentrated around 0, and the true coefficient (0.0243) is far away from these coefficients and is a clear outlier; furthermore, a large number of *p*-values are above 0.1. The results indicate that the core findings of this paper are robust.

#### Other robustness tests

The digital economy as suggested by the baseline results, significantly increases the financial market participation rate of Chinese households. However, so as to exclude confounding factors from the findings, further robustness tests are conducted in this paper. In this paper, we first consider that the explanatory variables increase simultaneously with the time trend; therefore, column (1) of Table 5 controls for the time trend for the regression. To prevent variations resulting from time trends in city-level characteristic variables from having an influence on the study findings, column (2) controls for the interaction term between city-level characteristics and time trends. Meanwhile, our research controls for the interaction term between urban fixed effects and time trends in column (3), and the estimates are all significant at the 1% statistical level. Second, so as to avoid confounding the findings by outliers on a larger scale, column (4) and column (5) are subjected to 99% and 95% tail shrinkage of the core variables, respectively, and the estimation results remain robust. Simultaneously, the study discovers via collecting and combing documents that the implementation of the "Notice on the Launch of National Smart City Pilot" policy during the sample period may impact the findings of this paper. For the purpose of avoiding the bias of the baseline regression results caused by the impact of other policies on the participation rate of Chinese household financial markets throughout the sample period, column (6) adds dummy variables of other policies. The study findings are excluded from the interference of other policies, and the estimated results after excluding policy interference are comparable to those of the benchmark regression. In addition, matching before double difference estimation may yield cleaner causality. We used nearest-neighbor matching within the caliper range, reserving the common support domain samples for estimation. Matching resulted in a lower difference in the probability distribution of propensity score values between the intervention and control groups. Column (7) reports the PSM-DID method regression results, which remain significant at the 1% level.

**Table 5:** Other robustness tests.

	FPR							
	(1)	(2)	(3)	(4)	(5)	(6)	(7)	(8)
Policy	0.024***	0.028***	0.031***	0.024***	0.024***	0.027***	0.023***	
	(0.005)	(0.005)	(0.008)	(0.005)	(0.005)	(0.005)	(0.006)	
$\widehat{Policy}$								0.046**
								(0.022)
SmartCity						-0.014		
						(0.110)		
Control	Yes	Yes	Yes	Yes	Yes	Yes	Yes	Yes
TimeTrend	Yes	No	No	No	No	No	No	No
TimeTrendxFeature	No	Yes	No	No	No	No	No	No
TimeTrendxCity	No	No	Yes	No	No	No	No	No
City FE	Yes	Yes	Yes	Yes	Yes	Yes	Yes	Yes
Year FE	Yes	Yes	Yes	Yes	Yes	Yes	Yes	Yes
N	686	686	686	686	686	686	545	547
r2_a	0.598	0.606	0.631	0.619	0.603	0.601	0.602	0.560

Standard errors in parentheses.

*p < .10, **p < .05, ***p < .01.

Although the previous results provide some evidence for the exogeneity of Broadband China's policies, this paper employs an instrumental variables approach to further validate the conclusions. We use historical postal and telecommunication data in 1984 for each city in China as the basis for constructing instrumental variables. On the one hand, the Internet is a continuation of the development of traditional communication technology, and the basic level of local historical communication facilities may affect whether a local area can be shortlisted as a pilot of the Broadband China policy; on the other hand, the impact of traditional communication tools, such as post offices and fixed-line telephones, on economic development diminishes with the decline in the frequency of use, which satisfies the exclusivity. It should be noted that the raw data of the instrumental variables selected are in cross-sectional form and cannot be directly used for the econometric analysis of panel data. In this paper, we introduce a time-varying variable to construct the panel instrumental variable^26^.

Specifically, the number of Internet users in the country in the previous year is constructed as an interaction term with the number of post offices and telephones per 10,000 people in each city in 1984 as an instrumental variable for the city's digital economy index in that year.Column (8) of Table 5 shows that the results are still significant after endogeneity is taken into account. In addition, for the test of the original hypothesis "insufficient identification of instrumental variables", the p-value of Kleibergen-Paap rk's LM statistic is 0.000, which is a significant rejection of the original hypothesis, and for the test of weak identification of instrumental variables, the Wald F-statistic of Kleibergen-Paap rk is greater than 10% for Stock-Yogo weak identification test. In the test of weak identification of instrumental variables, the Kleibergen-Paap rk Wald F statistic is greater than the critical value at the 10% level of the Stock-Yogo weak identification test. Overall, the above tests illustrate the rationality of selecting the cross term between the number of telephone sets in each city in history and the size of national Internet investment in the same year as the instrumental variable for the Internet index.

### Heterogeneous effects

From the objective investment environment, the level of development of the digital economy and the information environment in which China's urban and rural areas are located vary, and from the heterogeneity of households, the level of financial literacy also leads to different awareness of household investment, which affects household financial market participation rate. For examining the heterogeneous impact of the digital economy on the financial market participation rate of Chinese households, this paper takes two heterogeneous factors into account, urban–rural regional differences, and differences in household financial literacy, in further research so as to investigate whether there is heterogeneity in the impact of the digital economy on the financial market participation rate of Chinese households. This paper constructs financial literacy indicators from three dimensions: financial information, financial knowledge and financial capability, and calculates financial literacy scores using the iterative principal factor method. In this section, the paper uses the median score of financial literacy as a dividing line to delineate the high and low levels of financial literacy of households, Households with financial literacy scores greater than the median score are high financial literacy households, and households with financial literacy scores less than the median score are low financial literacy households.

The results in columns (1) and (2) of Table 6 depicts that the "Broadband China" pilot policy has increased the urban household financial market participation rate by 3.26%, nonetheless, the impact on the rural residents' household financial market participation rate is not significant. This is likely due to the uneven level of economic development between urban and rural areas in China, as well as the "digital divide" in the backward rural areas. The "digital divide" hinders the promotion of the digital economy on the rural household financial market participation rate. The results in columns (3) and (4) indicate that the "Broadband China" pilot policy increases the financial market participation rate of high financially literate households by 2.14%, but has little effect on the financial market participation rate of low financially literate households. This is probably because highly financially literate households are generally more active than low financially literate households in searching for relevant economic and financial information. The development of the digital economy is more likely to stimulate the desire of highly financially literate households to participate in financial markets, and the promotion effect on the financial market participation of low financially literate households is not pronounced.

**Table 6:** Heterogeneous effects.

	FPR			
	(1) Urban	(2) Rural	(3) Low financial literacy	(4) High financial literacy
Policy	0.0326***	0.0100	0.0093	0.0214***
	(0.0065)	(0.0125)	(0.0101)	(0.0049)
Control	Yes	Yes	Yes	Yes
City FE	Yes	Yes	Yes	Yes
Year FE	Yes	Yes	Yes	Yes
N	404	188	289	397
r2_a	0.4701	0.3015	0.4314	0.4812

Standard errors in parentheses.

*p < .10, **p < .05, ***p < .01.

### Mechanism tests

The development of digital infrastructure and digital media represented by the Internet is the core element of the growth of the digital economy, which acts on household financial market investment decisions by altering residents' information search channels. The paths for promoting household financial market participation are as follows: first, the information transmission path where the digital social network information transmission function is strengthened, the amount and efficiency of information content that households obtain through the social network are greatly enhanced, affecting household financial market participation behaviour; second, the financial information concern path, where the massive amount of Internet information makes households intentionally or unintentionally exposed to increased financial and economic knowledge as well as wealth management awareness. As a result, this paper uses the same two-step method to determine the proportion of households participating in the financial market in different cities after controlling for individual characteristics, based on the data of whether the respondents have smart Internet devices and financial information concerns and then regresses the interaction term of the "Broadband China" policy pilot. Of which, the respondents have intelligent Internet access devices as 1, the respondents do not have intelligent Internet access devices as 0; the respondents on the financial information concern level is based on the questionnaire data to the respondents on the financial information concern level of no concern at all to very concerned about the division of 1–5.

The regression results inTable 7 Column (1) points out that the digital economy has a significant positive impact on household ownership of smart Internet devices. In particular, the "Broadband China" policy pilot increases the total number of smart Internet devices held by households by 8.27%, and likewise considerably raises household financial information attention by 4.22% (Column (3)) , further influencing household financial market participation rate. Meanwhile Table 7 columns (2) and (4) show that the coefficient estimates of the core explanatory variables have decreased, and there is a partial effect of Home possession of smart Internet devices (SID) and Financial information attention (FIA) on the percentage of household participation in financial markets. mediating effect. In summary, the "Broadband China" policy is able to increase the proportion of households participating in financial markets by increasing the home possession of smart Internet devices (SID) and financial information attention (FIA).

**Table 7:** Mechanism tests.

	(1)	(2)	(3) (4)	
	Home possession of smart Internet devices(SID)	FPR	Financial information attention(FIA)	FPR
Policy	0.0827***	0.0232***	0.0422***	0.0215***
	(0.0160)	(0.0049)	(0.0073)	(0.0051)
SID		0.0390*		
		(0.0216)		
FIA				0.0592***
				(0.116)
Control	Yes	Yes	Yes	Yes
CityFE	Yes	Yes	Yes	Yes
YearFE	Yes	Yes	Yes	Yes
N	686	686	686	686
r2_a	0.4065	0.4521	0.4669	0.4815

Standard errors in parentheses.

*p < .10, **p < .05, ***p < .01.

## Conclusion

The conclusion that the digital economy greatly enhances household financial market participation remains valid after several robustness tests. The digital economy can further promote the household financial market participation rate by increasing the proportion of households holding smart Internet devices as well as the attention paid to household financial information, while the digital economy has a greater effect on the financial market participation rate of urban households and the financial market participation rate of households with high financial literacy. Hence, the government should pay considerable heed to the investment in network infrastructure construction in rural areas in order to reduce the distance between households and financial services in backward areas. This will enable the residents in backward areas to better enjoy the dividends brought by the development of the digital economy. Simultaneously, financial institutions and financial management departments should strengthen the popularisation of financial knowledge and enhance household financial literacy with the high permeability of digital media, so as to raise investors' awareness of risk prevention.

This paper highlights that the development of the digital economy may be a critical tool to solve the mystery of "limited participation" in the financial market of Chinese households, which is critical to effectively improve the financial welfare of Chinese households, raise the income of residents through multiple channels while enhancing people's sense of well-being.

### Supplementary Information

Supplementary Information.

## Data Availability

The datasets generated and/or analysed during the current study are available in the [CHFS] repository, [https://chfs.swufe.edu.cn].
