# Supplementary material for: Government digital policy breaks the mystery of “limited participation” in China's home finance market
Source: Sci Rep. 2023 Nov 19;13:20233. doi: 10.1038/s41598-023-47372-6 (PMC10658145; doi:10.1038/s41598-023-47372-6)
Supplement: Supplementary file 1 — Supplementary Information. [file 41598_2023_47372_MOESM1_ESM.docx]

**Appendix 1. Financial Literacy Principal Component Analysis Indicator System**

| Indicator | Specific questionnaires | Assign a value |
| --- | --- | --- |
| Financial information | Degree of concern for financial information | They are categorized as very concerned, concerned, generally concerned, rarely concerned and never concerned, and are assigned values from 5 to 1 respectively. |
| Financial knowledge | Calculation of interest rates | Respondent answered correctly = 1; Respondent answered incorrectly = 0 |
|  | Calculation of inflation rate | Respondent answered correctly = 1; Respondent answered incorrectly = 0 |
|  | The question of determining the Riskiness of Stocks and Funds | Respondent answered correctly = 1; Respondent answered incorrectly = 0 |
| Financial capacity | The question of risk appetite | Five bands of high risk and high return, slightly high risk and slightly high return, average risk and average return, slightly low risk and slightly low return, and unwillingness to take risk, with values ranging from 5 to 1. |
